# Editorial and Miscellaneous

**Published:** 1861-09

**Authors:** 


### EDITORIAL.

"*Put not your trust in*" *Local Editors.*—A medical contemporary is exceedingly exercised because, in a recent number, we quoted certain paragraphs from the columns of a prominent daily newspaper published in this city. We confess the soft impeachment and admit we credited its statements, largely because the acting editor of our medical contemporary is immediately identified with said daily newspaper, and therefore its utterances, in strict accordance with the "code," ought to be received as true.

We rejoice with our amiable contemporary in ascertaining that our public schools are not to be curtailed, or in anywise suffer in consequence of the delinquencies of its patron saint, for it appears said patron saint, fortunately, had nothing to do with the school moneys. It sufficiently appears that the individual from whom they derive the patronymic of their pet institution, only had to do with the *Sewerage* fund, and incidentally with the model medical college baptized with the promises he so liberally issued.

It leaks out that the model great medical reform school was endowed with a three year's lease of the cockloft of a warehouse and hide-and skin depot, provided the Faculty would put it in repair and good order, and were moreover incited to the work of medical reform by the express or implied assurance that their patron saint would erect them a new building wherein to inculcate medical reform, at the expiration of the three years' life and good behaviour, always provided it could be done with safety to the public funds, and without injury to the private resources of the patron saint.

There seems to be some strange contradiction in all this business. Our contemporary is Lind, and not Lind, according as the wind blows Northerly or Southerly. As secular editor, our contemporary attacks Mr. Lind; as medical editor, he defends Mr. Lind.

As secular editor, he insists that the patron saint has curtailed the fair proportions of the public schools; as medical editor, he resents the imputation with exceeding wrath. We are in doubt, therefore, and we anxiously inquire, is there any such thing as the Lind University, with academical and theological, and (grand climacteric) medical department, — "or any other man?"

Which way looks our contemporary? One of its editors abhors all that is fermented or distilled, and lifts up his voice in the highways and byways against it—the other is accounted such an acute judge of the virtues thereof, that the State Agricultural Society appoints him one of its judges of the excellence of ales, and silver pitchers incline to Sands, or Lill, or Dickinson, according to his refined taste. We despair of ascertaining the position.

But one thing has emerged from this contest—the Lind Medical College has no endowment, save promises now known to be impossible of fruition. Its assumed reformed methods of instruction have been repudiated by the whole body of the profession. It simply aims to build up a new school by misrepresentation of its own resources, and incessant calumny of the old. We leave it with its patron saint in the courts—the one to general medical opinion, the other the provided tribunals of justice under the Constitution and Statute Laws. We shall soon find which is the true Dromio.

---

*Books and Pamphlets Received.*—From Blanchard & Lea, through W. B. Keen, 148 Lake street, Chicago:

Bumstead on Venereal,

Barwell on Diseases of the Joints,

Morland on Uræmia.

Timely and valuable treatises, which will respectively receive early attention.

Transactions of the State Medical Society of Indiana; p. p. 56.

*Pott's Disease; or Angular Curvature of the Spine.* Cases successfully treated. By. J. A. Wood, M. D., Boston, Mass. Dr. Wood reports several cases of cure of this serious affection, "effected with the curvature nearly, if not completely removed, by appropriate mechanical appliances principally, to the entire exclusion of setons, issues, or any other counter irritant, or even restricting the patient to the recumbent posi tion. Without expressing any opinion further, we may observe that Dr. W. refers, by permission, to several of the most prominent surgeons in Boston and New York city, and can be consulted at his office, 31 Cooper Institute, N. Y. city. The apparatus employed has been described in one or more of the medical journals.

---

*Military Map and Gazetteer.*—Our military surgeons, as well as civilians, will find "Lloyd's Military Map and Gazetteer of the Southern Country" very convenient for a reference during the progress of the Grand Army toward the Gulf of Mexico. We acknowledge the receipt per mail of a specimen copy, upon which we hope to trace the daily progress of our brethren in the brigade and regimental service.

---

*Professional Patriotism.*—A private letter from one of the number, informs us that there are between 200 and 300 applicants for the position of Surgeon to the Tenth Regiment of Michigan Volunteers. Is there any other profession which can show proportionate zeal for serving the country? It is fervently hoped that all may not be forced to go.

---

*Rush Medical College.*—As noted in the last number of the Journal, there will be no interruption in the regular course of lectures the ensuing session, in consequence of the appointment of Profs. Blaney and Freer as Brigade Surgeons. These gentlemen will give complete courses as usual.

Prof. Blaney, having been selected as Medical Purveyor to the Illinois forces and Supervisor of the General Hospital department, is permanently stationed in this city. A letter from Prof. Freer, now in Washington, positively assures us that he will return and give his course, unabridged in any part.

The position of Demonstrator of Anatomy will, during the temporary absence of Dr. Powell, be supplied by the Prof. of Anatomy, assisted by I. P. Lynn, M. D., a well known and highly esteemed almunus of the College resident in this city.

The only modification of the usual curriculum will be the devotion of appropriate attention to Military Surgery and Practice, as rendered imperative by the exigencies of the times. It will be seen that, for carrying out this object, the College presents very considerable facilities; perhaps it is warrantable to assume, unequalled elsewhere.

---

*Oxide of Zinc.*—Dr. S. Waterman, in the *Medical Times*, asserts that Oxide of Zinc exerts a powerful sedative or soothing influence over the brain and the ganglionic nerves. It is mild in operation and free from the after effect of opium and other narcotics. It may thus be used in cases where other narcotics are contraindicated. " Its ability to soothe vascular excitement dependent on cerebral and nervous irritation cannot well be doubted; whilst it possesses equal power to allay and calm the irritation of the brain and ganglionic nerves, unaccompanied by inflammatory action of those tissues." He considers it even sometimes useful to a limited extent in inflammation of the brain and meninges. He concludes that both rationally and by actual trial and observation, it is beneficial in *delirium tremens*, when narcotics fail; given in two or three gr. doses every two hours, alone or in combination, as indicated. In *eclampsia infantilis*, after the fits have been broken by free use of chloroform; given in grain doses alone, or with cal. digit, or squill, &c. In *eclampsia*, happening during pregnancy, labor or menstrual irregularities; given in two or three gr. doses, every two hours. It is not as efficient in plethoric cases or uræmic intoxication. In *hysteric convul-*

*sions.* In *exanthematic diseases*, where the eruption accompanied by cerebral irritation or even convulsions. In *epilepsy* and *chorea.* In *asthmatic difficulties* and *whooping cough.* In the *night sweats* of phthisis. In *worms* and *chronic dysentery*, and lastly in some of the more obstinate cases of *intermittent.*

Dr. W. calls for further investigations of its properties.

---

*Gargle in Diphtheria.*—Common salt is strongly recommended as a gargle in diphtheria.

---

*Pitting in Small Pox.*—Dr. Bell commends the wearing upon the face of masses of cotton wool saturated with *Linimentum Aquæ Calcis*, the whole covered with a handkerchief, leaving apertures for the eyes, nose and mouth. The dressing should be kept sedulously applied from the commencement of the eruption until convalescence is fully established.

---

*Antaphrodisiac.*—Bromide of Potassium is again coming into favor as an antaphrodisiac. Pfeiffer, of Paris, confirms the opinion that it is a sedative both to the muscular and secreting portions of the generative apparatus. It appears to control both erections and discharges, and relieves neuralgia of the neck of the bladder and testicle. It is exhibited in moderate but repeated doses, every three or six hours.

---

*Rupture of the Abdominal Parietes.*—Edward Batwell, M. D., of Detroit, reports a case wherein the patient, a woman of 67, of rather intemperate habits, whilst sitting in her chair during a violent fit of coughing, had a complete rupture of the abdominal parietes "not alone through the muscular fibre of the recti muscles and linea alba, *but through the integument itself*, permitting a large quantity of omentum and small intestines to escape from their natural cavity, which were, with considerable difficulty, returned, owing to the strong muscular contraction and to the expression of pain from the patient."

"The rupture had occurred about an inch and a-half below the umbilicus, was about four inches long externally and about two inches in extent through the peritoneum. The recti muscles were severed through their entire breadth, and there was no appearance of ulceration or previous abrasion of surface, to account for the accident." The patient lived twenty-five days after receipt of the injury, the wound showing little or no tendency to unite. The case is certainly unique, but its credibility is most unquestionable.

---

*Chloroform in Tetanus.*—A correspondent (Dr. Wagenseller) of "Reporter," chronicling a case of idiopathic tetanus, unsuccessfully treated by chloroform, nevertheless concludes:

"1st. Chloroform does not always depress an already enfeebled circulation; although in this patient it acted the part of a powerful *nervous sedative*, it fulfilled at the same time the part of an *arterial stimulant.* This effect was *invariable;* these two important indications were met by it at every occasion of its administration.

"2d. Chloroform fully and freely administered will relax partially and temporarily the most persistent tetanic spasm, when opium and other remedies (which were tried in large doses) will produce no effect whatever."

He confidently looks to more recoveries from the use of this agent, than from any other now in use with the profession.

---

*Nervous System.*—In a recent lecture on the Spinal Cord, Bernard urges that the functions of the sympathetic nerve and cerebro spinal system so long viewed as entirely different in their nature—organic life being the sphere of activity of the first and animal life of the second—are by no means separable by absolute distinction. That the terms "sympathetic nerve" and "cerebro-spinal system" ought, with many others of like nature, to be expunged. There is no essential physiological distinction. The ganglia are not independent sources of nervous action. The results of experiments all tend to prove that all the branches of the sympathetic are derived from various regions of the spinal cord. The difference in the mode of action and conduct of the several nerves is not sufficient to establish a real diversity of nature.

He concludes: "The nervous system, therefore, contains only two great divisions—sensitive and motor nerves; it matters little whether the subject is conscious of their action or not, their properties in both cases being identically the same."

We have not space for further details from this interesting paper, (translated by Dr. L. Elsberg,) but must express gratification that this acute observer is gradually receiving the idea of the beautiful simplicity of the mechanism of nervous action. One step further is demanded, viz: the nervous system contains *no* divisions. The conduct of a nervous fibre, either as a sensitory or motory, depends *not on the fibre itself*, *but upon the structure which it reaches at its extremity.* Nerve fibre, wherever it is distributed, is simply a conductor determining changes at one extremity which have been originated, in its course, or, at the other extremity. The ultimate effect may be sensory, motory, &c., or, in one word which includes all *secretory*, according to the structure of the tissue or organ reached. The essential effect is a modification of the nutrition of the part reached, and hence necessarily a modification of its functional actions.

---

*Didn't See the Point.*—Some wit, chagrined at the failure of the distinguished authoress, Mrs. Ellis, to comprehend what he supposed to be a good perpetration, indignantly exclaimed that she could not see a joke if it was fired out of a cannon. What was his dismay when she with great astonishment inquired, "How *could* a joke be fired out of a cannon?"

Something like this occurred at Washington the other day, when the qualifications of a candidate for Brigade Surgeon were being looked over. A venerable member of the Board asked whether the candidate had *operated* much. Certainly, was the grave response,—he has operated extensively—*in real*

*estate.* The venerable examiner, with a puzzled air, remarked that he could not see *the analogy.* The candidate, however, was duly commissioned.

---

*Renunciation of Homœopathy.*—Dr. J. C. Peters, of N. Y. city, whilom distinguished as an author, editor and practitioner of the homœopathic school, is out in the *Am. Med. Times* with a letter renouncing and denouncing that system of delusion and imposture. We understand that several other prominent homœopathists of that city have followed his example. This is honest and honorable. But up here in the Northwest things are done differently. After very close observation and frequent conversation with homœopathists, we have been unable to find one who adheres to his assumed system. We have detected, time and again, their use of the usual doses and methods of regular practitioners. Many of them, indeed, make a merit of their readiness in "severe cases" to have recourse to "old school measures." The gross inconsistency, not to say dishonesty, of this course is apparent. But what is the use of arguing the matter? A certain proportion of the populace will have a pet delusion or imposture of some sort, and, perhaps, in its way, this absurdity of homœopathy is as harmless to them as any.

---

*Corps of Medical Cadets.*—We have received several letters of inquiry as to the proposed Corps of Medical Cadets. As we possess no particular information upon the subject beyond what is contained in the following circular from the Surgeon-General U. S. A., we insert it in full:

"Surgeon-General's Office, August 6, 1861.

"The following Act of Congress in relation to the Corps of Medical Cadets is published for the information of all concerned:

"'Sec. 7. *And be it further enacted*, That there be added to the Medical Staff of the Army a Corps of Medical Cadets, whose duty it shall be to act as dressers in the general hospitals and as ambulance attendants in the field, under the direction and control of the medical officers alone. They shall have the same rank and pay as the military Cadets at West Point. Their number shall be regulated by the exigencies of service, at no time to exceed fifty. It shall be composed of young men of liberal education, students of medicine, between the ages of eighteen and twenty-three, who have been reading medicine for two years and have attended at least one course of lectures in a medical college. They shall enlist for one year, and be subject to the rules and articles of war. On the fifteenth day of the last month of their service, the near approach of their discharge shall be reported to the Surgeon-General, in order, if desired, that they may be relieved by another detail of applicants.'

"Application must be made to the Surgeon-General for admission into the corps, in conformity with the above act, stating the date and place of birth, place of residence, period of medical studies, and enclosing the certificate of the dean of the college (or, when not attainable, other satisfactory evidence of the fact), that the applicant had attended one full course in a medical college. Those applications must also be accompanied with testimonials of the good moral character and sound physical condition of the candidate.

"When an applicant is favorably considered, the candidate will receive a letter authorizing him to appear before an Army Board of Medical Examiners, who will make a special report in such case. From among those approved by the Board the Surgeon-General will select such a number as the service may require.

"As the services of this class of medical and surgical assistants are at once required, applications (to be successful) should be promptly made to the Surgeon-General, who will direct the candidate to appear before one of the Army Medical Boards now in session in Washington and the city of New York.

"R. C. Wood, Acting Surg.-Gen."

---

*Perils of the Service to the Medical Staff.*—Whilst upon the field, Surgeons, like other non-combatants, are exposed to the chances of an occasional stray bullet, shot or shell. In the heat of conflict, but especially during a rout, they are in more or less danger from the ignorant brutality of a pursuing soldiery; but this latter is a rare and exceptional case. The highly wrought stories of Confederate butcheries of surgeons and wounded soldiers, repeated after the Bull Run affair, have, we are happy to believe, been almost wholly disproved.

Dr. Alfred Powell, Surgeon of the Second New York Regiment, was unquestionably killed while engaged in succor of the wounded, but there appears no evidence that his professional character was known, or that the catastrophe was more than one of the incidental brutalities of even the best conducted *christian* and *humane* wars. The surgeons who were taken prisoners generally concur in the statement of the kind treatment of the enemy, towards themselves, as well as the wounded under their care. The principal trouble seems to be the danger that, in the absence of the usual system of exchange of military prisoners, they are in danger of being retained in the enemy's custody, or placed on a parole, which will debar them for further profiting by their successful trial before the Examining Board.

But if surgeons, as in some instances reported, so far forgot their non-combatant character as to seize sword or musket and act the fighting part, of course they must take the soldier's chances. So even the wounded, if, through motives of either revenge or patriotism, they still continue, as in many cases related, to load and fire upon the enemy, they must not expect the ordinary immunity of the disabled soldier, but to render themselves liable to bayonet or ball. For the honor of the Anglo-Saxon race, we shall continue to believe that the killing of surgeons or wounded men, thus far reported, has at least this palliation. Meanwhile we shall continue to believe that the position of Surgeon to a Regiment, even if not particularly lucrative, or especially prolific of honor or reputation, at all events is not so remarkably dangerous as to frighten from it men of ordinary strength of nerve.

---

*The Rank.*—The vexed question is apparently settled by official information from the Adjutant General's office. The Regimental Surgeon of the Volunteer force, ranks and draws pay as a Major of Cavalry; the Assistant, as Captain of

Cavalry. This is better than feared; but now let the Government vouchsafe, brevets, promotion and the usual honorary mention of those who distinguish themselves in this service, and the Surgeons of the Grand Army will have little of which to complain. The importance of the department demands at least this.

---

*Iron Pyrites.*—Some correspondent, whose letter unfortunately has been mislaid, encloses a fragment of iron pyrites which a hopeful friend has handed him. Gold occurs in connection with this pyrites in gold bearing regions, but not elsewhere. Tons of this pyrites are worthless. The gold, if present at all, is in homœopathic attenuation, anything but pleasant to the miner, however much a similar attenuation may titillate the palate of the devout believer in infinitesimals. Homœopathy is Small in this city, but in value about equivalent to the gold in our friend's pyrites.

---

*A few Words about Typhoid Fever.*—All the phenomena of Typhoid fever tend to convince us that it is essentially dependent upon a *materies morbi* in the blood. The results of treatment illustrate it, and we take for granted that it is as well established as possible without its being absolutely sequestered and demonstrated by the chemist.

A large amount of this *materies* unattended by efforts at elimination, involves the phenomena of Cerebral Typhus. A large amount and excessive effort at elimination induces the graver forms of Typhoid (Abdominal Typhus); a lesser amount and active excretion presents the more common mild forms.

It is quite unfortunate that under the general term, "inflammation," are grouped a great variety of very different conditions. The term, in fact, is as indefinite as that of "fever." The intestinal affection in this disease is too frequently considered and treated as an imflammation, whereas it is no more nearly allied to common inflammation, than the small pox pustule and its surroundings are to the *tumor*, *rubor*, *calor* and *dolor* succeeding a healthy wound.

We are invited in practice to imitate the mode adopted by the *vis medicatrix naturæ* in endeavoring to eradicate disease. A better plan is to watch the tendency to death, and provide against it by such measures as scientific experience may suggest.

The tendency to death in the greatest portion of cases which have fallen under our notice has been by ulceration, hæmorrhage or perforation in the ileo-cœcal region. In every case examined, *post mortem*, the Peyer's patches have been involved, as sufficiently described in the books.

None have recovered after perforation—three cases. Three-fourths have recovered after hæmorrhage—eight cases. With these exceptions the disease has proved very amenable to treatment, and the results satisfactory.

We must be permitted to say that the so-called complications, gastritis, pneumonitis, meningitis, &c., have been infrequent under our observation, though frequently our professional friends have insisted that certain symptoms proved their existence. If these various organs were inflamed, the inflammation was of such a character as to merit quite a different name and treatment. In our view, too much ammunition has been wasted on "complications," especially by the book-writers. They might as well devote their batteries to the gouty toe, and neglect the gouty carcase.

Nothing is more true than the statement that in Typhoid fever, while there is life the case is not hopeless; and while there is any disease, there is danger. In the first case of perforation of the bowel in our practice, the patient had so far convalesced as to be up and about, and there was absolutely no external or rational indication of remaining disease; and yet, while at stool, the pains of perforation came on and he sunk, and was dead in six hours. On the other hand, we have had patients apparently moribund recover, in very spite of the prognosis.

The diagnosis is easier made than given. We rely upon the group of symptoms in the outset, and the roseolar eruption later as pathognomonic.

I. Languor, lassitude, mental hebetude, diarrhœa, epistaxis, continued fever, slight bronchial cough.

II. Roseolar Eruption—*always* present if carefully looked for, tympanites, sudamina, deafness, dryness and redness of the tongue, gurgling in the ileo-cœcal region (often with tenderness) upon pressure, (French method), nervous symptoms, delirium, subsultus, &c.

III. Gradual convalescence. Or, deepening of previous symptoms to constant delirium, coma, involuntary discharges, retention of the urine, prostration, &c., &c.

Average duration of treatment, nineteen days. A few cases will spin out to four, or even six weeks.

Treatment.—No emetics. No blood-letting. No active cathartics, except in the cerebral form with torpid bowels—*rare*.

First importance—quiet sleep at night. For this purpose a single full dose of Pulv. Ipecac. Comp. is preferred. The cases are very few when a large dose of this will not secure sleep, provided the patient does not fancy he has an idiosyncrasy with reference to opiates. (*Mem.*—The less a patient thinks he knows about medicine, the more likely he is to be benefited.) Rarely some other narcotic may prove useful.

Second point—that the bowels move once a day, and no oftener. To check the diarrhœa, Pulv. Opii and Acet. Plumbi in moderate doses, p. r. n. To move the bowels, Ol. Ricini vel Olivæ with twenty or thirty drops of Sp. Terebinth. Practically, in this disease, all saline cathartics are objectionable.

Third item—control febrile action by external sponging, cool air, free ventilation, cooling diuretics and diaphoretics. The diuretics are particularly preferred. Especially Acetas Potassæ: 1st, because it is practically useful by experience; 2d, because it eliminates a larger amount of solid matter through the urine than any other known article. For instance this:

R. Acet. Potassæ dr. ij—oz. ss.: Sp. Nitri. Dul. oz. ss.:
Pot. Tart. Antim. gr. ij.: Aq. oz. iij. ss.
M. Two teaspoonfulls every three hours.

If it inclines to purge, add Tr. Opi. Camph. q. s. or Tr. Opi. The hobby of the hour, Tr. Verat. Virid., may be substituted for the Pot. Tart. Antim., either in or out of the mixture, for its known effects. In our opinion, it controls symptoms well, but shortens no disease. It is hence infinitely inferior to the Antimonial. It is more liable to leave a red tongue and augmented intestinal disorder. It is neither restorative nor depurative, except in dangerous doses. In perhaps the majority of instances we rely upon the simple solution of the Acet. Potassæ, without adjuvants. Later in the disease Liq. Ammon Acet. is substituted occasionally. In this connection, we beg leave especially to recommend free and constant use of the Acet. Potassæ. No other diuretic or diaphoretic has anything like its power as a febrifuge and eliminating agent in continued fever. We cannot but believe, from long and close observation of its effects, that it relieves the system of that material which, in tending to excretion by the ileocœcal region, so prominently deranges and destroys the glands and other structures of that part.

It lessens thirst and thus obviates the necessity so often felt of deluging the stomach and bowels with diluents, eminently nastly decoctions and shadowy soups. Hence diarrhœa lessens, and tympanites with the accompanying train of nervous symptoms abates. There will be less need of Valerian, and Assafœtida, with its after effect of horrible odors.

Fourth proposition—When the red tongue, especially if dry and shining, appears, our experience adds nothing to the instructions of Prof. Wood as to the employment of the Ol. Terebinth, except a most cordial and hearty endorsement. It is *the* treatment—and the *ne plus ultra* of satisfactory treatment. In a very few cases, it would not be borne, and these cases have responded readily to moderate doses of mercurials—not carried to salivation. With this latter exception, we have found no use for mercurials in typhoid fever.

Fifth—As "a looker-on in Vernon" we have seen alcoholic stimulants used freely in the Typhoid fever, but must enter protest against them as a mode applicable to rustic constitutions. The cachectic crew that throng the fever wards of metropolitan hospitals furnish no standard by which country practice is to be guided—especially as the results here would speedily drive the practitioner from his "field of labor." Let it be remembered, the object of administration of the preparations of the alcohol is to arouse and invigorate the digestive and assimilative processes—not to relieve the abnormal feelings of weakness, or muscular and mental hebetude.

In the vast proportion of cases they are unnecessary and as hurtful as they are to the healthy man. Their abstraction, when once commenced with, produces proportionately the same effects as upon the habitual tippler. Considering the terrible consequences attendant upon the use of alcohol, it is the duty of medical men to avoid its prescription except in cases clearly and distinctly demanding it. It is of comparatively trivial value in the disorders now under consideration, and liable to the production of serious mischief. It will allay the nervous symptoms, it is true, but is not unlikely to aggravate the intestinal ulceration. At the very best, it is noticeable that those who rely upon it, have very sick patients and a multiplicity of "complications."

*Patients rarely die of simple debility;* the reason of death is deeper, and we have no evidence, theoretical or practical, that alcohol searches it out. A much more useful stimulant is furnished in Carbonate of Ammonia. Aristol. Serpent. is also very frequently quite serviceable. The stimulus they afford is more akin to the natural forces, and beyond this they do not gorge the blood with carbon. But in the—

Sixth note—Appropriate diet is beyond all other means the real sustaining power. Concentrated nutriment is easier digested than attenuated dilutions. Dish-water slops will rumble along the canal and so outward, in one perpetual flood. Nourishment should be communicated so as not to require mastication, but should be retained in the mouth long enough to become fully insalivated. This simple thing alone will moisten many dry tongues. While there is much activity of febrile action, it is better to eat nothing; then farinaceous boluses, (not slops); then strong essence of beef, (not "beef tea"); then hashed beef; then such meat as the appetite craves and the teeth are willing to thoroughly masticate, commingled with bread, &c., up to ordinary diet.

There is no objection to some condiments, as black pepper and salt. A table-spoonful of essence of beef has more supporting power than the same quantity of brandy; but dilute it with slops, and it *will* go with the "chocolate."

When the blood begins to show signs of impoverishment, not only must nitrogenized animal food be freely administered, but there will be a saving of alcohol and vital power by mingling a generous proportion of animal fat. It is fearful to think of the number who have died of inanition, whilst some old crone, or old crone's adviser, has been assiduously skimming off the scattered globules of oil from the boiled shadows of the valetudinary *soupe maigre*. Sick diet! *Lucus a non lucendo!*

Seventh particular—The drinks should never go beyond what will be speedily absorbed from the mouth, throat and stomach. The patient will rarely suffer from thirst if treated freely with the salt above indicated.

Eighth; last, but not least—Free ventilation is all important. Poor people recover in the country better than rich ones; because the healthful air and salutiferous light are not excluded. No place outside of a hospital (the charnel house of typhoid fever) is worse for the patients than the close, heavily curtained, heavily carpeted, and cumbrously furnished rooms of the affluent. No patients are so subject to pulmonary complications as those who are carefully guarded from "taking cold." A feather bed is herein an abomination. The entire paraphernalia of the couch should be changed daily. One day for service and one day out of doors. The evacuations are pestilential—out of doors with them *instanter*. The emanations are pestiferous—dilute them with outer air and sunlight. Have nothing in the room more than twenty-four hours in succession which is capable of absorbing effluvia.

Do not keep out the blessed sunlight or pure air. Put on a cheerful visage, even if playing Sir Oracle. The awakening and sustaining pleasant emotions in the patient's mind, is worth more than a gallon of "Huxham."

---

*A Practical Treatise on Military Surgery.*—By Frank H. Hamilton, M. D., New York. Baillière Brothers, 1861, pp. 234, 8vo.

This is the third work on military surgery whose appearance we have had occasion to chronicle since the commencement of the present war.*

Although this work of Prof. Hamilton takes a wider range than the others, being a "treatise," while those of Drs. Tripler and Blackman, and that of Dr. Gross, have only the modest title of hand-book, yet they all embrace essentially the same field and aim to supply the want supposed to be felt by surgeons called suddenly from civil practice to the duties of the field or military hospital. As such, they are all calculated to be useful. None of them assume to discuss the doubtful questions of surgical practice from an original point of view or in the light of any new facts.

It is indeed obvious that we most look to the end rather than to the beginning of any war for those works which can add something of value to our surgical knowledge, and, therefore, we have not heretofore, nor do we propose now to enter into any discussion of doubtful questions. The present work of

* For notice of the works of Drs. Tripler and Blackman, and that of Dr. Gross see page 308 of JOURNAL.

Prof. Hamilton embraces many subjects of interest as may be gathered from the table of "contents" subjoined :

Chap. I—Introduction ; II—Examination of Recruits ; III —General Hygiene of Troops ; IV—Bivouac, Accommodation of Troops in Tents, Barracks, Billets, Huts, etc. ; V—Hospitals ; VI—Preparations for the Field ; VII—Hygienic Management of Troops upon the March ; VIII—Conveyance of Sick and Wounded Soldiers ; IX—Gunshot Wounds ; X—Amputations ; XI—On the Employment of Anæsthetics in Amputations and other Surgical Operations, after Gunshot Injuries ; XII—Hospital Gangrene ; XIII—Dysentery ; XIV —Scorbutus, or Scurvy ; Appendix.

Of all these, we only feel called upon to caution all surgeons against accepting without reserve the views enunciated in regard to the use of anæsthetics, which the author regards as injurious in operations for severe wounds, and quotes Drs. Z. Pitcher and J. B. Porter, U. S. A., and Messrs. Velpeau, Alcock and Jobert in support of his views. It is regarded as tending to produce gangrene and prevent union by the first intention.

The objections urged against chloroform we regard as not so much against its use as its abuse, for we have often witnessed in some of the large hospitals of Europe and this country, its employment to an extent apparently unnecessary, if not dangerous. Our author prefers sul ether as an anæsthetic. After commencing with ether, going to chloroform and resorting to a mixture of the two, we have settled down upon the use of chloroform and in its constant employment in private and hospital practice for the last eight years, not a single case has occurred in which any unfavorable effect has resulted from its use.

As this is a point of great importance, and the opinions and experience of Prof. Hamilton and others *seem* to conflict with our own, the following points concerning its use are presented as rendering it entirely safe :

1. Let the patient be lying down.

2. Do not give it where there is disease of the heart or brain, or when the respiration is imperfect.

3. Administer it slowly on a coarse napkin, and let the air of the room be fresh and not charged with the vapor.

4. It should not be given when the stomach is full, but if the patient be depressed, stimulants may be administered before hand.

5. Its effects should be carefully watched, so as not to carry it too far. Interruption of the respiration, pallor or blueness of the lips, stertor and irregular pulse are indications for suspending its use. When the patient no longer shows signs of consciousness when questioned, the effect is sufficient.

If these hints be observed, chloroform is safe, and although the patient will show signs of pain during the operation, he will have no recollection of it afterwards.

The chapter on camp dysentery, by Prof. Austin Flint, is very judicious and will repay a perusal by those in civil practice.

The Appendix contains a recruiting of miscellaneous items of interest to those in or desiring to enter the military service.

Prof. Hamilton is now in the military service near Washington, and has published some letters of interest relating to the health of the camp, etc. He is in a position to be useful to the profession as well as to the country, and the results of his extensive experience will be eagerly anticipated by the profession. B.

